# Does elevated CO_2_ alter silica uptake in trees?

**DOI:** 10.3389/fpls.2014.00793

**Published:** 2015-01-13

**Authors:** Robinson W. Fulweiler, Timothy J. Maguire, Joanna C. Carey, Adrien C. Finzi

**Affiliations:** ^1^Department of Earth and the Environment, Boston UniversityBoston, MA, USA; ^2^Department of Biology, Boston UniversityBoston, MA, USA; ^3^The Ecosystems Center, Marine Biological LaboratoryWoods Hole, MA, USA

**Keywords:** elevated CO_2_, silicon, forest Si uptake, terrestrial Si pump, active Si accumulation, Si cycling

## Abstract

Human activities have greatly altered global carbon (C) and Nitrogen (N) cycling. In fact, atmospheric concentrations of carbon dioxide (CO_2_) have increased 40% over the last century and the amount of N cycling in the biosphere has more than doubled. In an effort to understand how plants will respond to continued global CO_2_ fertilization, long-term free-air CO_2_ enrichment experiments have been conducted at sites around the globe. Here we examine how atmospheric CO_2_ enrichment and N fertilization affects the uptake of silicon (Si) in the Duke Forest, North Carolina, a stand dominated by *Pinus taeda* (loblolly pine), and five hardwood species. Specifically, we measured foliar biogenic silica concentrations in five deciduous and one coniferous species across three treatments: CO_2_ enrichment, N enrichment, and N and CO_2_ enrichment. We found no consistent trends in foliar Si concentration under elevated CO_2_, N fertilization, or combined elevated CO_2_ and N fertilization. However, two-thirds of the tree species studied here have Si foliar concentrations greater than well-known Si accumulators, such as grasses. Based on net primary production values and aboveground Si concentrations in these trees, we calculated forest Si uptake rates under control and elevated CO_2_ concentrations. Due largely to increased primary production, elevated CO_2_ enhanced the magnitude of Si uptake between 20 and 26%, likely intensifying the terrestrial silica pump. This uptake of Si by forests has important implications for Si export from terrestrial systems, with the potential to impact C sequestration and higher trophic levels in downstream ecosystems.

## INTRODUCTION

We are currently conducting a global experiment by exposing Earth’s biosphere to atmospheric carbon dioxide (CO_2_) concentrations unseen since the early Miocene, some 23 million years ago (Pearson and Palmer, 2000). From the start of the Industrial Revolution we have increased CO_2_ concentrations by approximately 40% (Pearson and Palmer, 2000) and in the spring of 2014 CO_2_ levels officially exceeded 400 ppm at the Mauna Loa Observatory (NOAA, 2014). This grand experiment will continue for the foreseeable future, as over the next century CO_2_ concentrations are expected to increase further (IPCC, 2013).

The impact of increased CO_2_ concentrations on the terrestrial biosphere has received much research attention over the last several decades. In particular, recent research has focused on how plants have and will respond to rapid CO_2_ concentration increases in combination with other regional and global climate changes, including warming air temperature and increased N availability (Norby and Luo, 2004; Ainsworth and Long, 2005; Bernhardt et al., 2006; Finzi et al., 2006). Among numerous other impacts, CO_2_ enrichment can also alter plant stoichiometry (Loladze, 2002). Exposure to elevated CO_2_ concentrations can cause declines in leaf nutrients, such as N and phosphorus, as well as trace element concentrations (Taub et al., 2008). The mechanism driving this decline is unclear but it has been attributed to nutrient limitation, increased non-structural carbohydrates, lower transpiration rates, or changes in nutrient allocation patterns (Roberntz and Linder, 1999). A compilation of studies on herbaceous and woody plants found that elevated CO_2_ concentrations decreased foliar element concentrations by up to 15% (Loladze, 2002). Research on rice, a key global crop, found similar declines in essential elements such as N, magnesium, and iron. Such declines in plant elemental concentrations could have serious repercussions for higher trophic levels, including exacerbation of human malnutrition (Loladze, 2002). A meta-analysis by Cotrufo et al. (1998a) found that aboveground N content declined on average by 14% under high CO_2_ concentrations. Changes in the N content of litter have also been shown to alter rates of decomposition and thus nutrient cycling within terrestrial ecosystems (Cotrufo et al., 1998b). However, changes in foliar elemental composition could also have large-scale impacts on the cycling and transport of nutrients from land to the sea.

Silicon (Si) is the seventh-most-abundant element in the universe and the second-most abundant element in soils, the mineral substrate for most of terrestrial plant life (Epstein, 1994; Tréguer and De La Rocha, 2013). In the ocean, Si is a key nutrient required for diatoms and is used by many species of sponges, radiolarians, silicoflagellates, choanoflagellates, and even picocyanobacteria (Baines et al., 2012). Of particular importance are diatoms, as they form the base of many productive marine food webs and they sequester significant amounts of C to the deep ocean. In fact, a recent modeling effort contributes 50% of global ocean productivity to diatoms (Rousseaux and Gregg, 2013). The primary source of Si to the ocean is the transport of dissolved and biogenic silica (BSi; SiO_2_) via rivers, which together account for 78% of the net annual Si oceanic inputs (Tréguer and De La Rocha, 2013). The Si transported by rivers ultimately comes from the weathering of the lithosphere, which is dependent on complex interactions between climate, geology, and biology (Bluth and Kump, 1994; Conley, 2002; Derry et al., 2005). Recently there has been emphasis on the role of biology in altering the timing and magnitude of Si export, specifically in terms of biological uptake by terrestrial vegetation (Conley, 2002; Fulweiler and Nixon, 2005; Gérard et al., 2008; Carey and Fulweiler, 2013b) and the role of human activities in directly altering watershed Si export (Clymans et al., 2011; Carey and Fulweiler, 2012a, 2013b; Vandevenne et al., 2012).

Plants readily absorb dissolved silica (DSi), also known as silicic acid (H_4_SiO_4_), the dominant form of Si in soil solutions (Epstein, 1994). DSi is taken up with water and carried in the transpiration stream where, with the evaporation of water, it becomes supersaturated and precipitated as BSi or phytoliths (Raven, 2003). Si provides numerous benefits to vegetation including increased resistance to bacteria, fungi, and grazers, as well protection from desiccation and metal toxicity (Hodson and Evans, 1995; Epstein, 1999; Wieczorek et al., 2014). Si is found throughout plants, from their roots to their shoots, but peak concentrations are generally observed at the transpiration termini (Canny, 1990). In fact, Si can compose 10% or more of the dry weight, exceeding those concentrations of well-known macronutrients (i.e., N and potassium; Epstein, 1994). In turn, the accumulation of Si by terrestrial vegetation over the seasonal cycle has the capacity to regulate the watershed export of Si to coastal ecosystems as plants grow and senesce (Fulweiler and Nixon, 2005; Carey and Fulweiler, 2013b). Alternatively, because BSi is 7–20 times more soluble than mineral silicates (Cornelis et al., 2010a), plants may also provide an important source of Si on biological times scales.

Within this context we wanted to determine if trees exposed to elevated CO_2_ concentrations would exhibit a decline in foliar Si content like those observed for other elements. To do this, we analyzed BSi concentrations of leaf samples from coniferous and deciduous trees from the Duke free-air CO_2_ enrichment (FACE) experiments in North Carolina. Additionally, we examined Si content under nitrogen (N) enrichment, as well as under the combined impact of CO_2_ and N enrichment. This is the first study to specifically examine the role of CO_2_ and N enrichment on Si content in trees.

## MATERIALS AND METHODS

The Duke Face experiment is located in a *Pinus taeda* L. (*P. taeda*, loblolly pine) plantation at Duke University in North Carolina (35°58′N, 79°06′W). This plantation was established in 1983 and is characterized as having moderately low-fertility and acidic clay loam (McCarthy et al., 2010). In addition to the dominant pine, deciduous species present include *Acer rubrum* (*A. rubrum*, red maple), *Cercis canadensis* (*C. Canadensis*, red bud), *Cornus florida* (*C. florida*, dogwood), *Liquidambar styraciflua* (*L. styraciflua*, sweet gum), and *Ulmus alata* (*U. alata*, winged Elm). Mean annual precipitation is 1145 mm.

This study was conducted on leaves collected from CO_2_ and N enrichment experiments that took place between 1996 and 2006. The technical details of these studies have been published previously (e.g., Andrews et al., 2000; Luo et al., 2003; Finzi et al., 2007; McCarthy et al., 2010). Briefly, triplicate 30 m diameter treatment plots were exposed to current +200 ppm of CO_2_ above ambient during daylight hours in the growing season. Control plots (*n* = 3) were treated in a similar manner but with the addition of ambient air instead of CO_2_ (McCarthy et al., 2010). In 1998, two of these plots were divided in half and N fertilization began (11.2 g N m^-2^ y^-1^ as urea). For more detailed information on the experimental design see .

For this analysis we used dried green leaf samples from *P. taeda* and the five broadleaf species listed above, collected in 2002–2003 and 2006, respectively. *P. taeda* samples (*n* = 31) were separated and ground into a powder using a mortar and pestle. The pre-milled deciduous samples (*n* = 98) each contained composites of several individuals.

Biogenic Si concentrations were determined using a wet alkaline chemical extraction in a 1% Na_2_CO_3_ solution (Demaster, 1981; Conley and Schelske, 2001). Duplicate samples were weighed to approximately 30 mg (between 28 and 34 mg) and digested in flat bottomed polyethylene bottles in a shaker bath at 85°C and 100 rpm for four hours. We used a Seal AA3 flow injection autoanalyzer to colorimetrically determine DSi from the BSi aliquots using the molybdenum blue colorimetric method (Strickland and Parsons, 1972). Standards made of sodium hexafluorosilicate (Na_2_SiF_6_) as well as external standards were used throughout the analysis to check accuracy and were always within 4% of the expected value. We report all BSi values as %Si by dry weight.

All statistical analyses were completed using JMP Pro 10.0 and significance was judged with an alpha of 0.05. BSi concentrations across species and treatments exhibited equal variances according to several commonly used unequal variance tests (O’Brien = 0.6385, Brown-Forsythe = 0.3196, Levene = 0.1978). To explore potential drivers of BSi concentrations we used a linear mixed effects model to address if the BSi concentrations were different across species and treatment alone and combined. In this model we treated station as a random effect to assess the potential random station effects of the block design used at the Duke FACE experiment. We used an ANOVA to further explore differences in BSi concentrations across species and followed it by a *post hoc* means comparison with Tukey’s test for honestly significant differences (HSD).

## RESULTS

**FIGURE 1:**
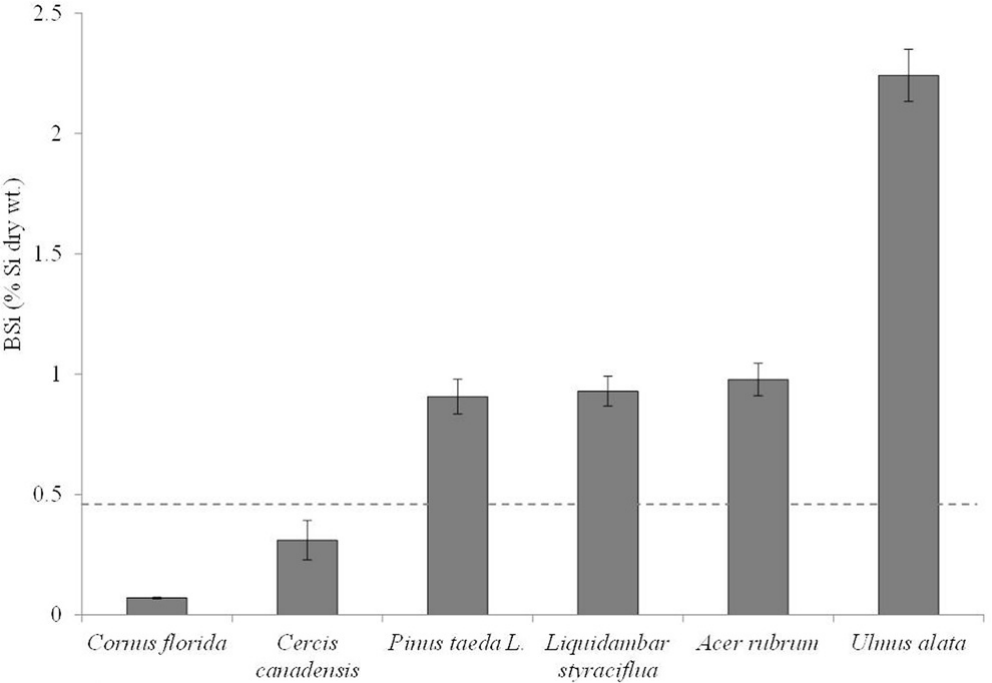
**Mean BSi concentrations (as %Si by dry wt.) in ascending order for the species analyzed in this study.** The most simplistic definition of active Si accumulation in plants is defined as above ground %Si > 0.46 which is shown here with the dashed line.

Across all sample types and treatments BSi values ranged from 0.05 to 3.01 %Si dry wt., with a median of 0.82 %Si dry wt. and mean of 0.98 %Si dry wt. *C. florida* exhibited the lowest and least variable BSi concentrations, while *U. alata* had the highest (**Table 1**; **Figure 1**). While some species did exhibit a decline in foliar BSi concentrations under elevated CO_2,_ we found no statistically significant effect of treatment on foliar BSi concentrations (**Table 1**). In fact, our least squared model showed no effect of treatments, station, or treatment by species.

**Table 1:** The mean foliar biogenic silica (BSi) concentrations (%Si by dry wt. ± SE) under the three treatment types and the control for five deciduous and one coniferous species at the Duke FACE experimental forest.

Treatment				
Species	Control	CO_2_ enrichment	N enrichment	N and CO_2_ enrichment
	(%BSi as Si dry wt.)			
*Cornus florida* (dogwood)	0.07 (±0.01)	0.07 (±0.01)	0.07 (±0.01)	0.08 (±0.01)
*Pinus taeda* (loblolly pine)	0.97 (±0.07)	0.90 (±0.36)	nm	nm
*Cercis canadensis* (red bud)	0.27 (±0.02)	0.19 (±0.00)	0.16 (±0.02)	0.50 (±0.21)
*Acer rubrum* (red maple)	1.06 (±0.12)	0.91 (±0.08)	0.98 (±0.19)	0.94 (±0.06)
*Liquidambar styraciflua* (sweet gum)	1.08 (±0.16)	0.95 (±0.05)	0.72 (±0.03)	0.92 (±0.11)
*Ulmus alata* (winged elm)	2.36 (±0.16)	2.57 (±0.23)	2.17 (±0.11)	1.90 (±0.35)

*nm = not measured.*

Because of the lack of statistical difference in plant BSi concentrations across all experimental treatments, we averaged BSi concentrations by species. Clear species differences exist (**Figure 1**), as the majority of species are statistically different from one another (*p* < 0.0001). The exceptions are that *P. taeda*, *A. rubrum*, and *L. styraciflua* are not statistically different from each other, but they are statistically different from *C. florida, C. canadensis*, and *U. alata*. *U. alata* exhibited BSi concentrations statistically higher than all other species (*p* < 0.0001). In all cases, except for *C.* and *C. canadensis*, the mean Si foliar content suggests active accumulation (i.e., %Si above 0.46%; **Figure 1**).

## DISCUSSION

In this study we examined foliar BSi concentrations in five deciduous and one coniferous species across three treatments: CO_2_ enrichment, N enrichment, and N and CO_2_ enrichment. We expected to see a decrease in Si content as CO_2_ increased, compared to the control group, as this phenomenon has been observed for a range of other elements such as N, phosphorus, iron, and zinc (Loladze, 2002). In four species (*P. taeda, C. canadensis, A. rubrum, L. styraciflua*) we did observe a decline in BSi concentrations under elevated CO_2_ but none of them were statistically significant (**Table 1**). The largest decrease of 35% was observed in *C. canadensis* while the others ranged from 7 to 15%. These declines are on par with those reported for essential elements in other plant species and thus, the lack of significance may simply be due to a limited sample number. In one species, *U. alata*, we observed a small, non-significant increase in BSi concentrations under CO_2_ enrichment (**Table 1**). We know of only one other study that examined the impact of elevated CO_2_ on plant BSi concentrations. In that study of cotton (*Gossypium hirsutum* L.cv. Deltapine 77), the Si concentrations increased by 26%, although the results were also not significant (Huluka et al., 1994).

We observed a decline in BSi concentrations in N enriched trees compared to the control ranging from 8 to 50%, but none were statistically significant (**Table 1**). Similarly, no significant differences between BSi concentration in CO_2_ enriched vs. N enriched were found for any of the species. The combined impact of N and CO_2_ enrichment again exhibited mixed results. For three species (*A. rubrum, L. styraciflua, U. alata*) we observed BSi declines from 12 to 21% and in one species (*C. canadensis*) we observed an increase of 60% (**Table 1**). Previous work at this same site found no significant differences in leaf N, phosphorus, C, lignin, or total non-structural carbohydrates under elevated CO_2_ (Finzi et al., 2001). This is in contrast to other studies that found decreases in N and increases in C concentrations in other tree species (quaking aspen, *Populus tremuloides* and paper birch, *Betula papyrifera*; Lindroth et al., 2001). Elevated CO_2_ concentrations also decreased concentrations of N, potassium, phosphorus, and sulfur in Norway spruce (*Picea abies* L. Karst; Roberntz and Linder, 1999). From these and many other studies, foliar chemistry response to elevated CO_2_ and N appears to be site-specific. Additionally, although we did not observe statistically significant effects, the trends we document may be ecologically important. For example, Si provides defense against herbivory and the 15% decline we observed in *A. rubrum* could impact feeding preferences of vertebrate and invertebrate consumers. The lack of significance may be in part driven by our sample numbers and thus, a larger study with more data may produce different results.

Silicon uptake by plants is divided into three broad categories (active, passive, or rejective). In active accumulation, plants acquire more Si than they would through water uptake alone. In rejective accumulation, also known as excluder accumulation, Si is taken up at a slower rate than water. Finally, in passive accumulation, water and Si have similar uptake rates (Raven, 1983; Ma et al., 2001b). Plants are assigned one of these categories according to various definitions, a thorough discussion of which is beyond the scope of this paper, but see Carey and Fulweiler (2014) in this Special Issue for a full description. Briefly, previous definitions have been based on Si concentrations in aboveground tissues, on the ratio of Si to calcium, and on the relationship between porewater Si concentration and aboveground tissue Si concentrations (e.g., Jones and Handreck, 1967; Takahashi et al., 1990; Ma et al., 2001a, respectively). More recent work has focused on the presence/absence of Si transporter genes in roots (*Lsi1* and *Lsi2*) and shoots (*Lsi6*) of rice (Ma et al., 2008; Yamaji et al., 2008). Research on accumulation modes in trees is surprisingly lacking. Cornelis et al. (2010b) observed both passive and rejective Si accumulation growth in coniferous tree saplings grown hydroponically. And a decrease in Si concentration with depth was observed in a temperate coniferous forest and designated as active accumulation (Gérard et al., 2008). Adult trees likely rely more heavily on groundwater and thus, in order to precisely determine the mode of Si accumulation, measurements of Si concentrations in groundwater, porewater, and within the tree are needed. Unfortunately, this is beyond the scope of this paper. Therefore, we apply the simplest definition that describes accumulation status as a function of Si concentration in the aboveground tissue alone: active accumulators as >0.46% Si by wt., passive accumulators as between 0.25 and 0.46% Si by wt., and excluders as <0.25% Si by wt. (Ma et al., 2001b; Street-Perrott and Barker, 2008). We acknowledge the limitations of this definition, as aboveground BSi tissue concentrations can be impacted by numerous factors, such as porewater DSi availability and external stressors. However, given our dataset, it is the one most appropriate for us to use. Defining Si accumulation status in trees by linking Si concentrations in aboveground vegetation to changes in porewater and groundwater, and locating Si transporter genes within trees are important areas of future research.

We observed a wide range in foliar BSi concentrations (**Table 1**). The low Si concentrations found in *C. florida* may indicate that these trees excluded H_4_SiO_4_. Overall however, the BSi concentrations observed in the Duke forest are 40 to 150% higher than those previously reported for similar species (Hodson et al., 2005). One reason for higher concentrations found in the Duke Forest could be the different soil and climate in North Carolina compared to the majority of studies reported in Hodson et al. (2005), which were dominated by northern temperate field sites. Our samples could also have higher BSi concentrations because of the land use legacy at the Duke Face site. In 1983, just 13 years before these experiments started, the forest was cut, trunks were removed, and the remaining material was burned (Finzi et al., 2001). The impact of land use change and disturbance on Si cycling is an emerging topic. From what we currently know, greater DSi losses have been observed following deforestation (Likens et al., 1970; Conley et al., 2008).

The median and mean BSi concentration (0.81 and 0.98 %Si dry wt., respectively) of the species we studied here is higher than many well-known actively Si accumulating groups, including those found in grasses (*Poaceae*) and sedges (*Cyperaceae*; Jones and Handreck, 1967; Raven, 1983; Ma and Takahashi, 2002). Uptake by forest trees has been hypothesized as a mechanism responsible for both the clear seasonal cycle of Si concentrations in stream water (Fulweiler and Nixon, 2005) and the observation of forested watersheds exporting significantly less Si than watersheds dominated by urban-land uses (Carey and Fulweiler, 2012a, 2013b). Given these data, and the known limitations of Si accumulation definitions, we can only hypothesize that this forest is actively accumulating Si. Regardless, the high Si concentrations we observed support the idea that forests are a critical component in regulating the flux of Si from land to the sea.

### DUKE FACE Si UPTAKE

**FIGURE 2:**
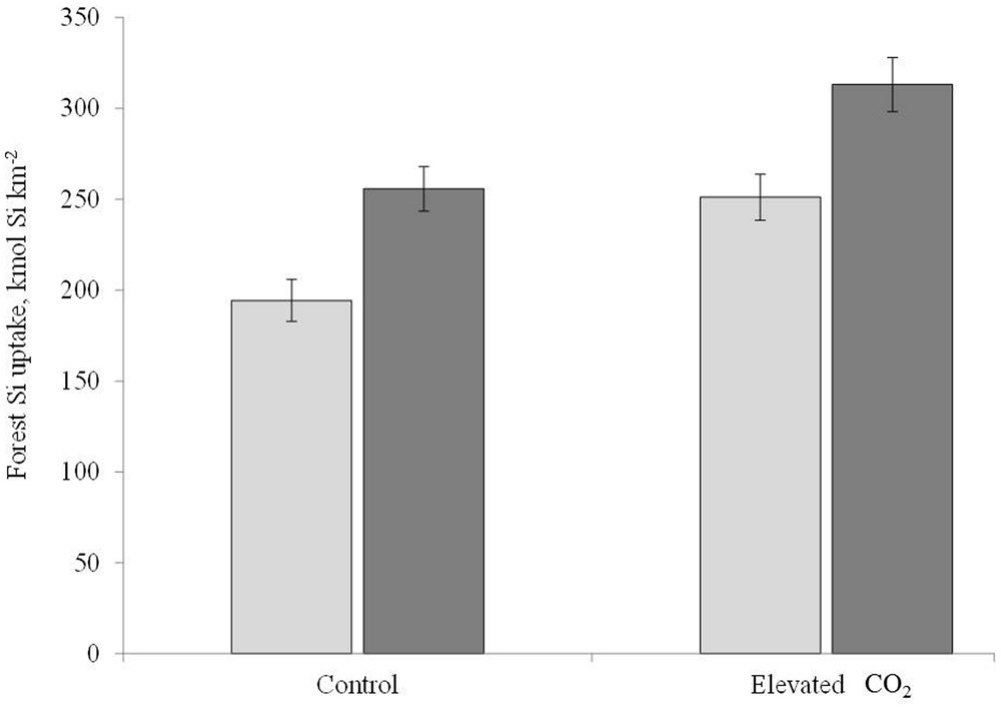
**A comparison between the control and elevated CO_**2**_ treatment of forest Si accumulation for the loblolly pine alone (light gray) and for all the species together (dark gray)**.

The impact of elevated CO_2_, N, and combined elevated CO_2_ and N led to an approximately 28% increase in net primary production (NPP) at the Duke Face site between 1996 and 2004 (McCarthy et al., 2010). We used the mean NPP over this period to estimate the amount of Si taken up by the Duke forest for the control and elevated CO_2_ treatments. We focused on the CO_2_ treatment because we do not have BSi concentrations for the *P. taeda* under N fertilization or the combined elevated CO_2_ and N fertilization treatments. Additionally, biomass at this site is dominated by *P. taeda*, which comprises ∼98% of the tree basal area (Finzi et al., 2001). We calculated foliar Si:C ratios by dividing the treatment specific median %Si value of either *P. taeda* alone or all the species together by 0.47, as C concentrations in biomass are well constrained between 45 and 50%. We then multiplied this Si:C ratio by the known amount of C in each treatment to get a treatment specific Si foliar uptake value (Conley, 2002; Carey and Fulweiler, 2012b). We estimated woody biomass Si uptake using the mean temperate woody biomass %Si of 0.08 (Fulweiler and Nixon, 2005) and again divided by 0.47. We then prorated the amount of Si by the proportion of leaves (30%) versus woody biomass (70%) in a typical forest to determine a total Si uptake rate (**Figure 2**; Litton et al., 2007). Although we observed a decline in Si concentrations under elevated CO_2_, the total amount of Si taken up by *P. taeda* was 26% higher at elevated CO_2_ where NPP was significantly higher compared to ambient CO_2_ (control: 194 kmol Si km^-2^, elevated CO_2_: 251 kmol Si km^-2^). Together with the hardwood species, Si uptake rate was 20% higher in the elevated compared to ambient CO_2_ treatment, although absolute rates of uptake were higher than *P. taeda* alone (elevated CO_2_ = 313 kmol km^-2^ y^-1^; ambient CO_2_ = 266 kmol Si km^-2^, **Figure 2**). These values are on the high end of those reported for forested systems but well within the reported range. For example, Gérard et al. (2008) reported 157 kmol Si km^-2^ for a Douglas fir forest in France, while Meunier et al. (1999) reported an uptake of over 3400 kmol Si km^-2^ in a bamboo forest.

A review of FACE experiments found that irrespective of ecosystem type, aboveground production increased in the presence of higher CO_2_ and trees were more responsive than herbaceous vegetation (Ward et al., 2013). Other studies have found similar increases in global terrestrial production (Melillo et al., 1993; Nemani et al., 2003). In addition, Pan et al. (2013) found that global forest biomass has increased in established forests. Such changes in primary productivity will also alter Si cycling. In fact, Si accumulating vegetation accounts for 55% of terrestrial NPP (33 Gton C y^-1^) an amount similar to the C sequestered by marine diatoms (Carey and Fulweiler, 2012b). This terrestrial Si pump has important implications for global climate, as Si cycling helps to control atmospheric CO_2_ concentrations through a variety of mechanisms, including chemical weathering of mineral silicates and C occlusion in soil phytoliths (Berner and Berner, 1997; Parr and Sullivan, 2005). In addition, this vegetation also plays a critical role in modulating the amount and timing of Si export from watersheds to downstream receiving waters (Carey and Fulweiler, 2012a, 2013b), which has direct implications for marine C dynamics. Here, we show that anthropogenically driven enhanced NPP may result in an increase in the terrestrial Si pump as forests take up more Si. In turn, the increased terrestrial Si sink may alter Si availability in aquatic systems. Diatoms require N and Si on a one to one molar basis (Redfield, 1963). Thus, the ratio of N to phosphorus to Si (N:P:Si) helps to control the composition and abundance of phytoplankton species assemblages. Human activities, such as fertilizer use and land use change have increased N and P loading to coastal systems worldwide. Phytoplankton respond to these elevated nutrients by increased productivity. At first, diatoms will bloom until all the Si is consumed at which point other non-Si requiring species will flourish (Anderson et al., 2002). The enhanced Si uptake by forests under elevated CO_2_ may be another way in which humans are altering nutrient stoichiometry in coastal receiving waters.

Missing from this discussion is the mechanism driving our findings that CO_2_ and/or N additions have no significant impact on Si accumulation in aboveground biomass. Of course, with more data and different study sites we might observe a significant impact of these factors on Si accumulation rates in forest or other terrestrial vegetation types. In fact, a recent study found higher Si concentrations in the porewater, sediment, roots, and occasionally the aboveground biomass of a heavily N enriched salt marsh (Carey and Fulweiler, 2013a). Certainly other factors such as changes in water availability and growing season length, as well as warming temperatures, are all factors that might influence foliar BSi accumulation. Here we propose an alternative idea for exploration: we hypothesize that in ecosystems with altered transpiration rates, corresponding changes to leaf Si concentrations will also be observed because Si is delivered to vegetation via water. Higher transpiration rates should, if passive or active accumulation is occurring, result in higher Si concentration in leaves, as Si is typically concentrated at transpiration termini. Conversely, if transpiration rates decline, then less Si will be deposited. Experimental work on Douglas fir saplings found higher Si concentrations that were attributed to greater transpiration in those seedlings (Cornelis et al., 2010b). However, at the Duke FACE experimental site no changes in transpiration were observed and maybe this is why we observed no significant changes in Si concentration (Ward et al., 2013). Complicating these simple ideas is the original hypothesis that motivated this work – enhanced CO_2_ lowers elemental composition of leaves such as N, phosphorus, and potassium. Thus, quantifying the interplay between structural changes in vegetation and transpiration rates, as well as water availability under future climate change scenarios, will be a critical next step in our understanding of climate change impacts on terrestrial Si cycling.

## CONCLUSION

Our data of BSi concentrations in 6 species from the Duke FACE Experiment does not support our initial hypothesis that elevated CO_2_ concentrations would decrease foliar Si content. In fact, we observed no consistent or significant impact of any of the treatments on foliar Si content. However, according to the simplest definition based on aboveground tissue Si concentrations, we did find evidence that four out of the six tree species we studied may be active Si accumulators. These tree species had Si concentrations higher than some of the most well-known Si accumulators (e.g., grasses and sedges). Further, the higher NPP values observed under elevated CO_2_ resulted in higher Si uptake rates under elevated CO_2_ conditions in the Duke forest. Based on this analysis we hypothesize that anthropogenic change, specifically elevated atmospheric CO_2_ concentrations, may increase biological Si pumping in forests, increasing the magnitude of the terrestrial Si pump.

## Conflict of Interest Statement

The authors declare that the research was conducted in the absence of any commercial or financial relationships that could be construed as a potential conflict of interest.
